# Responsible Relations: A Systematic Scoping Review of the Therapeutic Alliance in Text-Based Digital Psychotherapy

**DOI:** 10.3389/fdgth.2021.689750

**Published:** 2021-07-09

**Authors:** Charlotte M. van Lotringen, Lars Jeken, Gerben J. Westerhof, Peter M. ten Klooster, Saskia M. Kelders, Matthijs L. Noordzij

**Affiliations:** Department of Psychology, Health and Technology, University of Twente, Enschede, Netherlands

**Keywords:** therapeutic alliance, working alliance, text-based psychotherapy, internet-based psychotherapy, responsible digital health, mental health

## Abstract

**Introduction:** Developing a good therapeutic alliance is considered essential for the responsible delivery of psychotherapy. Text-based digital psychotherapy has become increasingly common, yet much remains unclear about the alliance and its importance for delivering mental health care via a digital format. To employ text-based digital therapies responsibly, more insight is needed into the type and strength of the therapeutic alliance online.

**Methods:** A systematic scoping review was performed searching four databases: Scopus, PsycINFO, Web of Science, and Wiley Online Library. A total of 23 studies were selected and data was extracted and tabulated to explore the characteristics of studies on text-based psychotherapy, measurements of the therapeutic alliance and associations of the alliance with treatment outcome.

**Results:** The therapeutic alliance in text-based digital interventions was studied with a variety of client groups, though mostly for clients diagnosed with anxiety and/or depression issues. Treatment modalities were predominantly internet-based cognitive behavioral therapy (ICBT) and tailored platforms for distinct client groups (e.g., PTSD). Almost all treatments used asynchronous text-based communication, such as e-mails and integrated messaging functions, which were mainly used to give feedback on tasks. For measurements, a version of the Working Alliance Inventory (WAI) was used in most studies. Measurements with the WAI or WAI- short form indicated a good therapeutic alliance with a weighted mean score of 5.66 (on a scale of 1 to 7) and a weighted standard deviation of 0.84. Relations between the therapeutic alliance and treatment outcomes were mostly positive, with many studies reporting significant associations (*n* = 8 out of 10) or significant effects of the therapeutic alliance on treatment outcomes (*n* = 5 out of 6).

**Discussion:** Our scoping review suggests that a good therapeutic alliance can be established in digital psychotherapy through text-based communication, and shows support for a positive relationship between the alliance and treatment outcomes. These findings illustrate that text-based online psychotherapy can be a responsible treatment option as far as the establishment of the therapeutic alliance is concerned. However, current measures of the therapeutic alliance might miss important aspects of the alliance in digital treatment, such as the presence of empathy or compassion.

## Introduction

The internet has long been explored as an alternative route to deliver psychological treatment. Benefits include easy, broad, and convenient accessibility and lowered costs (1). More specifically, internet-delivered psychotherapy can increase the availability of mental health care in underserved populations and during times of crisis (2), and could therefore provide a responsible alternative or addition to fully face-to-face therapy. Meta-analyses show moderate to large effects and comparable results of online therapy to face-to-face therapy in randomized controlled trials (RCT's) for a variety of psychopathological symptoms (3–5). Notwithstanding the evidence for similar effectiveness, many consider the relationship between therapist and clients to be a central component of successful psychotherapy, and its role online is questioned and not fully understood among researchers, practitioners and clients (6).

### Responsible Digital Treatment

In general, therapists do see multiple possible advantages of digital psychotherapy, such as new options for treatment and even increased intimacy in the therapeutic relationship (7). However, the higher accessibility of digital psychotherapy also gives them an increased sense of moral responsibility, especially in the case of crisis situations, where the lack of physical proximity might not allow the right caretaking actions. In those cases, the therapeutic alliance could be especially important to ensure that clients trust their therapists and share the issues that they may be facing, so that a response can be given promptly (8). In addition, therapists report a lack of information and confidence as some of the main barriers for their use of digital interventions (7, 9). Therefore, an important aspect of the responsible use of digital therapy is knowledge about the extent to which a good therapeutic alliance can be established in different digital treatment modalities.

### A Text-Based Alliance

One type of online psychotherapy where the role of the therapeutic relationship is still unclear is text-based digital psychotherapy. Text-based digital psychotherapy consists mainly of written exchanges via the internet, such as internet cognitive-behavioral therapy (ICBT) delivered *via* e-mail or chat (10). This form of digital psychotherapy has the potential to foster the therapeutic relationship in novel ways, for example by giving clients more time to reflect on the expression of their feelings and thoughts in written words, while not being observed. Similarly, therapists can benefit from the richness of the written word, and the additional time to reflect before responding (2, 11). Further, the lack of social cues encourages the development of alliances and contributes to higher levels of openness and self-disclosure (12). This could stimulate a close and strong relationship between clients and therapists in a different way than in conventional face-to-face therapy or videoconferencing therapy (13).

However, the lack of non-verbal cues in the text-based format can lead therapists to fear that the communication during therapy would be impaired, and raises concern as to whether a therapeutic alliance can develop through text-based online counseling (14). In addition, the question arises whether the type of alliance that does emerge is beneficial to the therapeutic work (10). To make responsible use of text-based digital psychotherapy, the aim of the current scoping review is to give a better understanding of the therapeutic alliance in digital, text-based communication, and its relation to therapy outcomes.

### The (Digital) Therapeutic Alliance

The therapeutic alliance is often operationalized through the concept of “working alliance.” The working alliance includes different collaborative aspects of the relationship between the therapist and the client (15). A highly influential model of the working alliance by Bordin (16) offers a pan-theoretical perspective on the relationship between therapist and client in therapy, with the core of the alliance being: agreement on therapy goals, agreement on therapy tasks and the bond between therapist and client. Therapeutic goals refer to the objectives of the therapy that are endorsed by both therapist and client. Tasks refer to the processes and behaviors in psychotherapy sessions that relate to the actual therapeutic work. The bond refers to the interpersonal attachment between therapist and client and should include confidence, acceptance and mutual trust (16).

Therapists rate the importance of the alliance in conventional face-to-face psychotherapy significantly higher than in online psychotherapy (17). Furthermore, therapists report less confidence in their abilities to develop a functional therapeutic alliance in internet-based psychotherapy (17). In contrast with this, research on internet-based cognitive behavior therapy suggests the quality of the therapeutic alliance, most commonly rated by the client, to be at least as strong as in face-to-face therapy and also highlights the association of the alliance with online treatment outcome (15, 18, 19).

More specifically, a meta-analytic review of the alliance in adult face-to-face and internet-based psychotherapy showed that the alliance was significantly related to treatment outcomes, with a similar association between alliance and outcome in face-to-face psychotherapy (*r* = 0.278) and internet-based psychotherapy (*r* = 0.275) (15). The positive relationship between the alliance and outcome appeared to be consistent across different alliance measures and outcome measures, treatment approaches (e.g., CBT, psychodynamic therapy, etc.) and client characteristics. Moreover, the overall correlation between alliance and outcome was almost identical to the one found in an earlier meta-analysis (20).

### Types of Digital Psychological Interventions

The efficacy of a broad range of digital psychological interventions has been demonstrated, as well as a similar association between alliance and outcome as reported in face-to-face therapy. However, many current reviews and meta-analyses on efficacy and alliance-outcome associations fail to differentiate between different types of psychological online interventions. The meta-analysis by Flückiger et al. (15), for example, combined the different types of e-mental health (via internet, e-mail, videoconferencing and phone). This limits our understanding and ability to make responsible choices between the various forms these interventions can take online.

One way to categorize online psychological interventions is offered by Berger and Andersson (21), who distinguish between modes of communication of psychological online interventions. Communication can be text-based and asynchronous (e.g., e-mail), text-based and almost real-time or synchronous (e.g., chat), and audio- or video-based synchronous communication (e.g., video-conferencing). The review by Berger (10) was the first to examine ratings of the working alliance using the categorization by Berger and Andersson (21). However, Berger's (10) review was narrative, and did not provide an overview of all the alliance measurements and relations to outcomes that studies reported. Moreover, compared to other forms of online interventions, Berger (10) found a very limited database on the alliance in text-based digital psychotherapy and called for more research on this therapy format. Therefore, the present review specifically explores the scope of currently available research on the therapeutic alliance in text-based internet psychotherapy as defined by Berger and Andersson (21), in which communication between the therapist and client takes place via the internet and is text-based (e.g., e-mail, chat). Since we are interested in the therapeutic relationship between the client and the therapist, studies examining unguided, self-help text-based digital interventions are not included here.

### Research Objective

The current scoping review gives a comprehensive overview of the nature and extent of current research evidence on the therapeutic alliance in text-based digital psychotherapy. This review maps out the study and intervention characteristics and findings of the existing research on this topic by exploring:

1. How text-based digital therapy is being studied: with what client groups and platforms, the forms, frequency and duration of text-based communication, the types of therapists and the treatment approaches.
2. What findings studies report regarding the working alliance: which measurements are used, what is the reported quality of the working alliance, and what types of statistical relationships between the therapeutic relationship and outcome of treatment are reported.

Our findings can indicate whether the strength of the therapeutic alliance in text-based digital psychotherapy is comparable to the one found in face-to-face psychotherapy, and if there is a relation with treatment outcomes. This way, the current review aims to enhance responsible decision-making in terms of the therapeutic alliance in digital text-based psychotherapy.

## Methods

### Research Design

The present literature review is a systematic scoping review, conducted according to the guidelines provided by Peters et al. (22). Scoping reviews intend to map out the current body of research on a specified topic in terms of nature, characteristics and volume. The assessment of the potential size and scope of available research is done systematically, transparently, and in order to be easily replicated (23). Systematic scoping reviews typically synthesize data into tabular form to summarize and disseminate the existing literature in the field of interest, to identify research gaps and to make recommendations for future research (22).

### Search Strategy

The electronic databases Scopus, PsycINFO, Web of Science, and Wiley Online Library were used to search for relevant studies published between 2005 and 2020. These databases were chosen because of their focus on social, medical and psychological topics, with PsycINFO being more narrowly focused on psychological and mental health research and Scopus and Web of Science being databases with a broader scope. Wiley Online Library was included because a first search of the literature indicated that many relevant articles stemmed from this database.

Each database was searched for articles and the search was repeated several times throughout the period of data collection to ensure an exhaustive and up-to-date evidence base. The final search was conducted on October 6, 2020. The databases were queried with the following combination of search terms (“working alliance” OR “therapeutic relationship” OR “therapeutic alliance”) AND (“internet-based psychotherapy” OR “online psychotherapy” OR “web- based psychotherapy” OR “online mental health”) occurring in the title, abstract or keywords of published scientific literature. The search term “text-based” was not included, since articles generally did not classify the therapy format in this way. Therefore, potentially relevant articles were scanned manually to evaluate whether the used digital psychotherapy format was indeed text-based.

### Eligibility Criteria

The following inclusion and exclusion criteria were established:

#### Inclusion Criteria

1. The article needed to describe original research (e.g., no literature review).
2. The language of the article needed to be in either English, German, Dutch or Spanish for an extensive review.
3. The year of publication had to be 2005 and onwards, as the technological context of the review topic makes it likely that studies from before 2005 are outdated.
4. Studies had to include text-based internet psychotherapy as defined by Berger (10).
5. Studies needed to include an assessment of the working alliance or similar construct using some validated measure, such as the Working Alliance Inventory (24).

#### Exclusion Criteria

1. Articles describing unguided internet-based self-help programs or interventions, video or audio-based internet psychotherapies, and internet-based psychotherapies that were offered in combination with face-to-face psychotherapy (blended treatment).
2. Articles that contained unclear descriptions of the interventions that they studied, making it impossible to determine if the accompanying therapeutic counseling was text-based.
3. Articles describing studies with participants without mental health issues (e.g. general health issues).

### Study Selection

**Figure 1:**
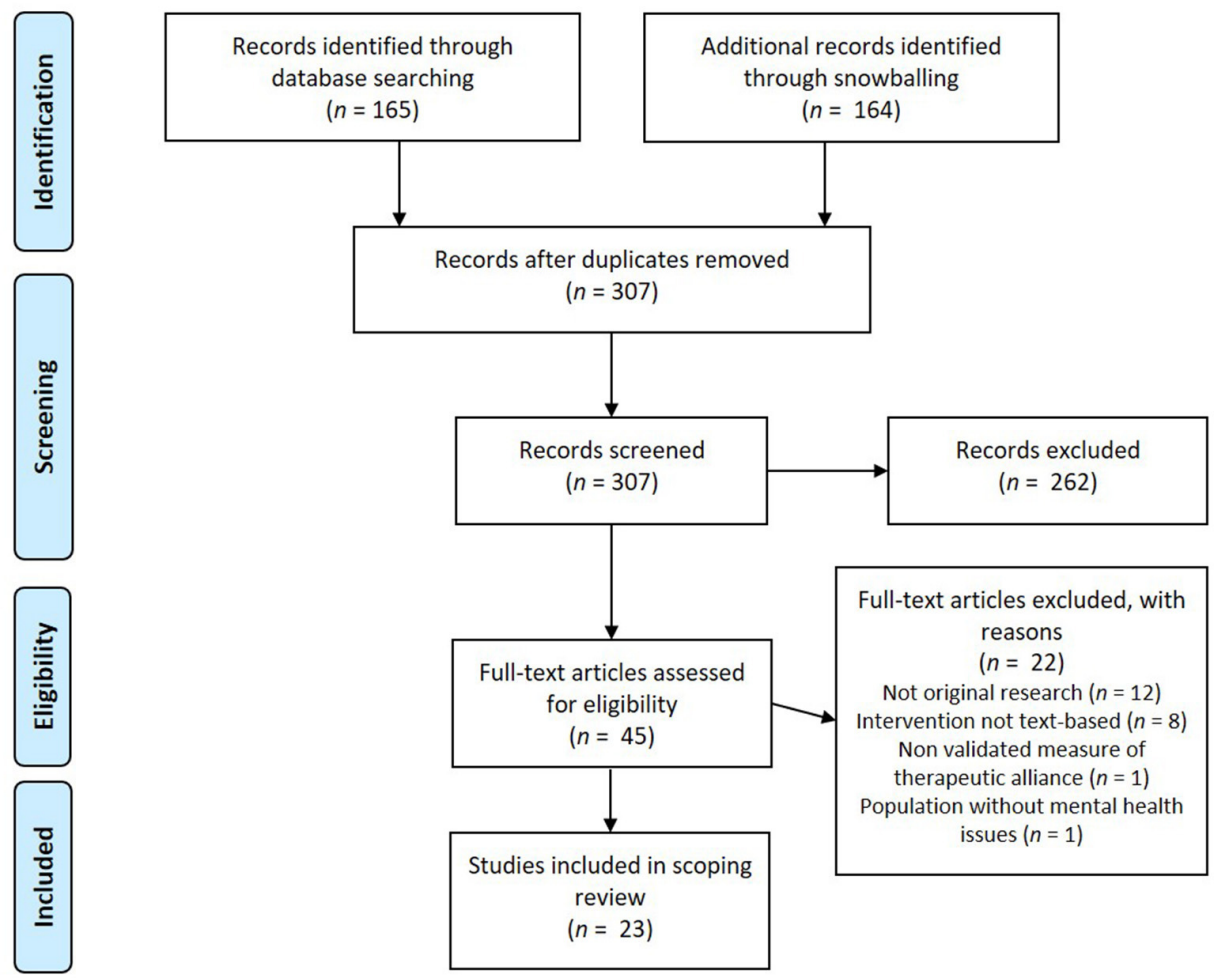
PRISMA flowchart (26) of the inclusion/exclusion process of articles for the systematic literature review.

Studies found were screened on title in the first step and on abstract in the second step by two authors (CML and LJ). In step 3 it was determined whether the remaining studies were eligible based on reading the full paper and eligibility was judged using the inclusion and exclusion criteria by two authors (CML and LJ). In both steps, disagreements were discussed between the authors until agreement was reached. Subsequently, the reference lists of the included studies, as well as the studies that cited the included studies were inspected [backward and forward snowballing; (25)] to find additional papers. A flowchart illustrating the study selection process of the present systematic literature review according to the PRISMA guidelines (26) is presented in Figure 1.

### Data Extraction

The collected articles of this review were fully read and analyzed in accordance with the aim of this research as specified above. All data from the selected articles were extracted by two researchers (CML and LJ). Data items that were extracted included participant characteristics, study and intervention characteristics and measures.

#### Participant Characteristics

Firstly, we extracted the targeted population: the mental disorders and/or problems that were treated. If an intervention targeted more than one disorder the group of disorders was described in accordance to the DSM-5 classification of mental disorders to describe the treated mental health problems targeted (e.g., the term anxiety disorders for several disorders including severe symptoms of anxiety, such as social anxiety disorder, general anxiety disorder, and panic disorder). Additionally, we extracted data on participants' gender and age.

#### Study and Intervention Characteristics

The study characteristics that were extracted were the study design and the sample size of participants. The intervention characteristics that were extracted included the platform and modalities used, the medium (e.g., mail, chat) and type of communication, the frequency of contact and average time spent by the therapist per client, the treatment length, the type of therapist, and the therapeutic approach the intervention was based on.

The type of communication within an intervention was categorized as synchronous or asynchronous, according to the categorization terms provided by Berger (10). To determine the type of practitioner, the exact wording in the study design section of the study was used. This was done to maintain the identity of the practitioner's group, as a grouping of these mental health professionals might bias the results, since different countries use different titles for various groups of mental health care workers (e.g., therapist, psychotherapist, psychological psychotherapist). If the therapeutic approach the treatment was based on was not specified by the researchers this was marked as “not specified.”

#### Measurements

We collected information about the instrument used to measure therapeutic relationship and the rater (client or therapist), the point in time of measurements, the reported quality of the working alliance, and if applicable, the reported statistical relationship between the therapeutic relationship and outcome measures. Cut-off scores for the categorization of the strength of associations were specified a-priori and based on the general guidelines by Cohen (27). Thus, a correlational value was labeled weak when below 0.10, small from 0.10 to 0.30, moderate from 0.30 to 0.50, and labeled strong from 0.50 to 1.00.

## Results

A total of 23 studies were reviewed for this scoping review, covering a total of 28 interventions. Among the included studies, 14 studies investigated the working alliance as a primary objective, while the other 9 studies assessed the concept as a secondary objective.

### Participant Characteristics

Table 1 summarizes the participant characteristics. Among the reviewed studies a variety of client groups was studied. The most commonly investigated client group were clients diagnosed with anxiety symptoms or disorders (*n* = 9). Another client group often assessed in working alliance research was clients diagnosed with depression (*n* = 8). Other investigated groups were clients diagnosed with posttraumatic stress disorder (PTSD) or PTSD-related symptoms (*n* = 6), obsessive-compulsive disorder (*n* = 2), binge-eating disorder (*n* = 1), preterm labor stress (*n* = 1), and chronic tinnitus (*n* = 1). In most studies the majority of participants were female, with the exception of one study having a low percentage of 39.5% female participants. The weighted average age across the studies was 37.4 years with a weighted average standard deviation of 8.77. Two studies investigated the working alliance in the treatment of children and adolescents and two studies the treatment of traumatized elderly.

**Table 1:** Participant characteristics.

	**References**	**Presenting problem**	**Gender**	**Age (years), mean (SD)**
1a	Andersson et al. (28)	Depression	75% female	38.9 (13.5)
1b	Andersson et al. (28)	Generalized anxiety disorder	80.6% female	40.0 (11.2)
1c	Andersson et al. (28)	Social anxiety disorder	59.3% female	37.7 (11.42)
2	Andersson et al. (29)	Obsessive-compulsive disorder	66.3% female	34.93 (12.72)
3a	Anderson et al. (30)	Anxiety disorders	61.6% female	13.91 (1.56)
3b	Anderson et al. (30)	Anxiety disorders	53.0% female	12.12 (2.5)
4	Bergman Nordgren et al. (31)	Anxiety disorders	67% female	39.3 (11.2)
5	Bisseling et al. (32)	Anxiety and depression during cancer	85.7% female	n.a.
6	Blake Buffini and Gordon (33)	I.a. mood, anxiety and personality disorders	83.3% female	n.a. (0.80)
7	Dölemeyer et al. (34)	Binge-eating disorder	93.2% female	34.8 (10.3)
8	Duffy et al. (35)	Depression and anxiety	69% female	n.a.
9	Penedo et al. (36)	Depression	70.4% female	44.48 (10.68)
10a	Hadjistavropoulos et al. (37)	Depression	69.5% female	40.22 (12.57)
10b	Hadjistavropoulos et al. (37)	Generalized anxiety	69.5% female	40.22 (12.57)
11	Herbst et al. (8)	Obsessive-compulsive disorder	n.a.	n.a.
12	Jasper et al. (38)	Chronic tinnitus	39.5% female	51.92 (10.55)
13	Klein et al. (39)	Posttraumatic stress disorder	77.27% female	66.1 (11.36)
14	Knaevelsrud et al. (40)	Posttraumatic stress symptoms	56.6% female	71.73 (4.8)
15	Knaevelsrud et al. (41)	Childhood traumatization	64.9% female	71.4 (4.7)
16	Knaevelsrud and Maercker (42)	Posttraumatic stress reactions	92% female	35 (n.a.)
17	Knaevelsrud and Maercker (43)	Posttraumatic stress disorder	90% female	35 (10.55)
18a	Lindegaard et al. (44)	Social anxiety disorder	62% female	41.4 (12.0)
18b	Lindegaard et al. (44)	Social anxiety disorder	74% female	42.6 (16.3)
19	Preschl et al. (45)	Depression	84% female	34.9 (9.5)
20	Reynolds et al. (46)	Mostly depression and stress/anxiety issues	71% female	n.a.
21	Scherer et al. (47)	Preterm labor stress	100% female	32.53 (3.49)
22	Topooco et al. (48)	Adolescent depression	91% female	17.5 (1.1)
23	Wagner et al. (49)	Posttraumatic stress symptoms	81% female	27.7 (7.0)

*If a study did not report certain information, this is indicated with n.a. (not available)*.

### Study and Intervention Characteristics

Table 2 summarizes the characteristics of the reviewed studies and interventions. Most studies had an RCT design (*n* =11), were part of a larger RCT study (*n* =1), or involved a pilot RCT (*n* = 1). Other study designs included controlled trials (*n* = 2) and open trials (*n* = 4). The sample sizes ranged from 13 to 223. Among the reviewed interventions all but one used internet-based modules as the modality. This modality uses treatment modules created by researchers or clinical psychologists for a specific target group, such as for PTSD (*n* = 3), binge eating disorder (*n* = 1), and stress management (*n* =1). The modules were accessible via the internet and allowed for communication between therapist and client, which mostly entailed feedback on writing tasks and progress in treatment. The exception was one study that used an instant messaging intervention, and did not specify the platform.

**Table 2:** Study and intervention characteristics.

	**References**	**Study design**	**Sample size**	**Modality**	**Form(s) of communication (asynchronous/synchronous)**	**Frequency of contact, average time spent by therapist per client**	**Treatment length**	**Therapists**	**Therapeutic approach**
1a	Andersson et al. (28)	Controlled trial	88	Internet-based modules	Either e-mails or feedback on self-help tasks (asynchronous)	Weekly, e-mail therapy: total of 509 min. (SD=176), guided self-help treatment: total of 53 min. (SD=28)	8 weeks	“Internet-therapist”	Cognitive behavior therapy
1b	Andersson et al. (28)	Controlled trial	89	Internet-based modules	Feedback on self-help tasks (asynchronous)	Weekly, 10-15 min. a week	8 weeks	“Internet-therapist”	Cognitive behavior therapy
1c	Andersson et al. (28)	Controlled trial	204	Internet-based modules	Feedback on self-help tasks (asynchronous)	Weekly, 15 min. a week	8 weeks	“Internet-therapist”	Cognitive behavior therapy
2	Andersson et al. (29)	RCT	101	Internet-based modules	Integrated text messaging function, emails (asynchronous)	2-3 times a week, n.a.	10 weeks	Clinical psychology students in their final year of the study program under supervision of a licensed psychologist	Cognitive behavior therapy
3a	Anderson et al. (30)	Controlled trial	73	BRAVE: Internet-based modules	Emails (asynchronous)	Weekly, n.a.	10 youth and five parent sessions	Registered psychologists	Cognitive behavior therapy
3b	Anderson et al. (30)	Controlled trial	132	BRAVE: Internet-based modules	Emails (asynchronous)	Weekly, n.a.	10 youth and five parent sessions	Registered psychologists	Cognitive behavior therapy
4	Bergman Nordgren et al. (31)	Randomized controlled pilot trial	27	Internet-based modules	Feedback on homework assignments (asynchronous)	N.a., 15 min. a week	10 weeks	Master's degree level psychology students who had completed clinical training	Cognitive behavior therapy
5	Bisseling et al. (32)	RCT	78	Internet-based modules	Written feedback on completed logfiles, emails (asynchronous)	Weekly, n.a.	9 sessions	Therapists fulfilling the advanced criteria of the Association of Minfulness-Based Teachers in the Netherlands and Flanders	Third wave CBT (Mindfulness-based cognitive therapy)
6	Blake Buffini and Gordon (33)	Cross-sectional	78	A service providing free online counseling during crises	Instant messaging (synchronous)	On demand, n.a.	Participants accessed support 2-5+ times	Service-staff with a minimum of undergraduate level in psychology, psychotherapy or social care	Not specified
7	Dölemeyer et al. (34)	Uncontrolled trial	59	Internet-based modules, based on ‘Overcoming Binge Eating’	Integrated text messaging function (asynchronous)	Weekly, n.a.	16 weeks	‘Therapist’	Cognitive behavior therapy
8	Duffy et al. (35)	Open, uncontrolled feasibility trial	123	SilverCloud: Internet-based modules	Written feedback on progress (asynchronous)	Every 10-14 days, n.a.	8 weeks	Clinical psychologists, counseling psychologists and psychological wellbeing practitioners	Cognitive behavior therapy
9	Gómez Penedo et al. (36)	RCT	223	Deprexis: Internet-based modules	Standardized email support with feedback on activity (asynchronous)	Weekly, n.a.	10 modules	Master's students in clinical psychology and psychotherapy, psychotherapists in training and licensed psychotherapists, trained in the program	Cognitive behavior therapy
10a	Hadjistavropoulos et al. (37)	Open dissemination trial	83	Internet-based modules	Feedback on homework, emails (asynchronous)	Weekly, n.a.	12 modules	Registered psychologists, social workers, nurses with CBT experience and supervised graduate students in clinical psychology or social work	Cognitive behavior therapy
10b	Hadjistavropoulos et al. (37)	Open dissemination trial	112	Internet-based modules	Feedback on homework, emails (asynchronous)	Weekly, n.a.	12 modules	Registered psychologists, social workers, nurses with CBT experience and supervised graduate students in clinical psychology or social work	Cognitive behavior therapy
11	Herbst et al. (16)	RCT	29	Internet-based modules	n.a.	n.a.	14 sessions	n.a.	Cognitive behavior therapy
12	Jasper et al. (38)	RCT	38	Internet-based modules	Online messaging systems (asynchronous)	Weekly, 13.75 min. per week	10 weeks	Clinical psychologists certified in CBT or psychologists in advanced stages of their training	Cognitive behavior therapy
13	Klein et al. (39)	Open trial	22	PTSD Online: Internet-based modules	Audio files and email individually tailored and constructed (asynchronous)	n.a., total time: 194.47 min. (SD=148.7)	10 weeks	Registered and probationary registered psychologists	Cognitive behavior therapy
14	Knaevelsrud et al. (40)	Open trial	30	Internet-based modules	Feedback on writing assignments (asynchronous)	n.a.	6 weeks	Doctoral-level clinician psychologists with special training in the application of CBT for PTSD	Cognitive behavior therapy and narrative exposure therapy
15	Knaevelsrud et al. (41)	RCT	94	Internet-based modules	Uploading texts in secure Web portal (asynchronous)	10 responses to texts, 45-50 min. each	6 weeks	Licensed clinical psychologists with special training in Integrative Testimonial Therapy	Cognitive behavior therapy
16	Knaevelsrud and Maercker (42)	Part of an RCT	91	Internet-based modules	Feedback on writing assignments (asynchronous)	n.a.	5 weeks	Psychologists trained in the application of writing assignments for PTSD	Not specified
17	Knaevelsrud and Maercker (43)	RCT	96	Internet-based modules	Feedback on writing assignments (asynchronous)	n.a.	5 weeks	Clinical psychologists trained in the application of writing assignments for PTSD	Cognitive behavior therapy
18a	Lindegaard et al. (44)	Preference study	13	SOFIE: Internet-based modules	Mail service within internet platform, feedback on homework assignments (asynchronous)	Weekly, guideline of 15 min. per week	10 weeks	Master's degree level psychology students	Cognitive behavior therapy
18b	Lindegaard et al. (44)	Preference study	23	Internet-based modules	Feedback on homework assignments (asynchronous)	Weekly, guideline of 15 min. per week	10 weeks	Master's degree level psychology students	Psychodynamic therapy
19	Preschl et al. (45)	RCT	25	Internet-based modules	Feedback on writing assignments and instructions for exercises (asynchronous)	n.a., 20-50 min. per text	8 weeks	Psychologists and psychotherapists trained in CBT for depression	Cognitive behavior therapy
20	Reynolds et al. (46)	Uncontrolled	17	Internet-based modules	Emails (asynchronous)	n.a.	n.a.	“Therapists, predominately qualified to practice in the US”	Not specified
21	Scherer et al. (47)	RCT	58	IB-CBSM: Internet-based modules for stress management	“Written exchange” (n.a.)	Weekly, n.a.	6 modules	Trained psychologist or psychologist in training	Cognitive behavior therapy
22	Topooco et al. (48)	RCT	70	Iterapi: Internet-based CBT with therapist chat sessions	Chat sessions conducted inside the treatment platform (synchronous)	Weekly, 45 min.	8 weeks	Therapists in training	Cognitive behavior therapy
23	Wagner et al. (49)	RCT	55	Internet-based modules for PTSD symptoms	Written feedback and instructions (asynchronous)	n.a., 20-50 min. per text	5 weeks	Psychologists and psychiatrists	Cognitive behavior therapy

*If a study did not report certain information, this is indicated with n.a. (not available)*.

Most reviewed interventions used asynchronous communication (*n* = 26), which involved feedback on self-help tasks and on written assignments, emails, and the use of an integrated text or chat function within the treatment platform. Text-based responses by a therapist were always created within 24–48 h after a message was sent by a client, questions were asked or writing assignments were completed. For one intervention, the type of communication was not specified and another reported “written exchange” without a detailed specification. Only two interventions (partially) used synchronous text-based communication involving a chat room used by the client and therapist.

The most common frequency of contact between the client and therapist for interventions was weekly (*n* = 16). Other frequencies that were used were 2–3 times a week (*n* = 2) and every 10–14 days (*n* = 1). One intervention for free online counseling was accessible on demand. For nine interventions, the contact frequency was not specified. For eleven of the interventions, the average time that was spent by the therapist per client was reported. The most common average time was around 15 min per client per week (*n* = 5). Interventions where the therapist replied to writing assignments indicated longer average times, from 20-50 min per client per text (*n* = 2) to 45–50 min (*n* =1). One of the two interventions that involved synchronous chat sessions reported weekly session durations of 45 min. The treatment length of the interventions ranged from 5 to 16 weeks, 6 to 10 modules or 9 to 14 sessions. For most included interventions, the treatment length was reported in weeks (*n* = 18), with a length of 8 (*n* = 6) or 10 weeks (*n* = 6) being the most common. One intervention that made use of instant messaging as a form of free online counseling, was accessed 2 to 5 or more times by participants.

In regard to the type of therapist offering or guiding treatment online, several interventions offered guidance by a licensed or registered psychotherapist or psychiatrist, and/or psychotherapists in training (*n* = 6), or psychologists, psychotherapists or psychiatrists of whom it was unclear if they were licensed or registered (*n* = 6). For other interventions, psychology or social work students in the final phase of their master's degree were (additionally) employed (*n* = 7). Among the reviewed studies some used the term “online therapist” or “therapist” to refer to their practitioners, without specifying the term further (*n* = 7), whereas one did not state who was responsible for communication with clients within the treatment program.

The majority of reviewed studies based their intervention on the cognitive behavioral therapy approach (*n* = 18). One study combined a cognitive behavioral therapy approach with narrative exposure therapy, one study used a mindfulness-based cognitive therapy approach, and another study used a psychodynamic treatment approach in one condition of their studied treatments. Finally, three studies did not specify which psychotherapeutic approach the treatment was based on.

### Measurements

Table 3 summarizes the findings concerning measurements of the therapeutic relationship and its relationship with outcome measures, with articles ordered by type of scale used.

**Table 3:** Measurements.

	**Authors**	**Therapeutic relationship measure and rater (c = client-rated, t = therapist-rated)**	**Moment of assessment**	**Quality of therapeutic relationship, Mean (Standard deviation)**	**Relationship between therapeutic relationship and outcome**
1a	Andersson et al. (28)	WAI: Working Alliance Inventory, C	Third week of treatment	5.41 (0.83)	Correlations between the WAI-S and residualized change scores on the primary outcome measures were weak (*r* = 0.18) and not statistically significant
1b	Andersson et al. (28)	WAI: Working Alliance Inventory, C	Third week of treatment	5.63 (0.94)	Correlations between the WAI-S and residualized change scores on the primary outcome measures were small (*r* = 0.13) and not statistically significant
1c	Andersson et al. (28)	WAI: Working Alliance Inventory, C	Fourth week of treatment	5.45 (1.05)	Correlations between the WAI-S and residualized change scores on the primary outcome measures were small (*r* = 0.10) and not statistically significant
2a	Anderson et al. (30)	WAI-S: Working Alliance Inventory-Short Form, C	After completion of third session	5.77 (1.20)	n.a.
2b	Anderson et al. (30)	WAI-S: Working Alliance Inventory-Short Form, C	After completion of third session	5.85 (1.09)	Higher WAI-S scores in older adults (12-18 years) predicted CGAS at 6-month follow-up (*B* = .22, *t* = 2.21, *p* = 0.03) Higher WAI-S scores predicted compliance with the treatment (*B* = 0.38), *F*_(1, 80)_ = 13.10, *p* = 0.01)
3	Blake Buffini and Gordon (33)	WAI-S: Working Alliance Inventory-Short Form, C	After participants had accessed support on more than one occasion	4.30 (1.27)	The strength of the working alliance predicted client satisfaction, explaining 55% of variance in client satisfaction scores (*R-square* = 0.55; *F* = 93.85, *p* < 0.001)
4	Dölemeyer et al. (34)	WAI-S: Working Alliance Inventory-Short Form, C	After first half of treatment	6.01 (0.79)	Correlations between the WAI-S measured at end of treatment and residual gain scores on EDE-Q-subscale ‘restrained eating behavior’ were significant and moderate (*r* = −492), no correlations between WAI-S and binge eating episodes
5	Knaevelsrud et al. (41)	WAI-S: Working Alliance Inventory-Short Form, C	End of treatment	6.2 (0.7)	n.a.
6	Knaevelsrud et al. (40)	WAI-S: Working Alliance Inventory-Short Form, C	Fourth treatment session	6.09 (0.87)	n.a.
7	Knaevelsrud and Maercker (42)	WAI-S: Working Alliance Inventory-Short Form, C & T (only client ratings reported)	Fourth treatment session	5.8 (0.62)	Correlations between the WAI-S and residual gain scores on anxiety were significant and moderate (*r* = .33)
8	Knaevelsrud and Maercker (43)	WAI-S: Working Alliance Inventory-Short Form, C & T	End of treatment	Client: 6.3 (0.54) Therapist: 5.8 (.98)	Correlations between the client-rated WAI-S (at the end of treatment) and treatment outcome were significant and predicted 15% of the variance in post-treatment measures of the IES-R (*adjusted R-square* = 0.148; *F*_(2, 39)_ = 8.15, *p* < 0.05)
9	Preschl et al. (45)	WAI-S: Working Alliance Inventory-Short Form, C & T	After four weeks	Client: 5.82 (0.80) Therapist (only measured post treatment): 6.04 (.67)	Correlations between clients' ratings of the subscale ‘tasks’ measured post-treatment and BDI-score at post-treatment in the online group were significant and moderate (*r* = −0.47), the WAI-S did not significantly predict the BDI residual gain score (*r* = −0.06)
10	Topooco et al. (48)	WAI-S: Working Alliance Inventory-Short Form, C	n.a.	4.95 (0.63)	n.a.
11	Wagner et al. (49)	WAI-S: Working Alliance Inventory-Short Form, C	After the fourth session	6.04 (0.83)	Early WAI-S (at mid-treatment) significantly predicted treatment outcome (*adjusted R-square* = .20; *F*(2,44) = 6.57, *p* = 0.003)
12	Andersson et al. (29)	WAI-S: Working Alliance Inventory-Short Form, n.a.	Third week of treatment	n.a.	Higher degree of working alliance predicted Y-BOCS change score (*B* = −0.09, *SE* = 0.04, *t* = 2.20, *p* < 0.05)
13	Bergman Nordgren et al. (31)	WAI-S: Working Alliance Inventory-Short Form, adapted for guided internet interventions, C	Third week	6.00 (0.80)	Correlations between the WAI-S (at week 3) and residual gain scores on the primary outcome measure were significant and moderate (*r* = −0.47)
14	Lindegaard et al. (44)	WAI-S: Working Alliance Inventory-Short Form, adapted for guided internet interventions, C	Third week	n.a.	Correlations between the WAI-S (at week three) and treatment outcome were significant and WAI-S predicted change rate (*B* = −0.05, 95% CI [−0.072, −0.018], *z* = −3.22, *p* = 0.001)
15	Bisseling et al. (32)	WAI-SR: Working Alliance Inventory-Short Form Revised, C	At the start of week 2	n.a.	Therapeutic alliance predicted both reduction of psychological distress (*B* = −0.12; *t*(114) = −2.656; *p* = 0.01) and increase of mental well-being (*B* = 0.23; *t*(113) = 2.651; *p* = 0.01) at post treatment
16	Herbst et al. (8)	WAI-SR: Working Alliance Inventory-Short Form Revised, n.a.	Post treatment	4.08 (0.78)	Correlations between the WAI-SR and Y-BOCS SR change score were significant and moderate (*r* = 0.33); a marginal correlation between WAI-SR and the OCI-R change score was significant and small (*r* = 0.29)
17	Jasper et al. (38)	WAI-SR: Working Alliance Inventory-Short Form Revised, C	Fifth week	2.34 (0.98)	Correlations between the subscales ‘agreement on treatment tasks’ and residual gain scores for the therapy outcome measure ‘tinnitus distress’ were significant and moderate (*r =* 0.40)
18	Scherer et al. (47)	WAI-SR: Working Alliance Inventory-Short Form Revised, adapted to the online help for women, C	After module 2, 3, 4, and 5	3.51 (.69)	Correlations of the WAI-SR and residual gain scores on stress and anxiety outcomes were significant and moderate (PSS: *r* = 0.451) and strong (STAI-T: *r* = 0.501). Nearly 40% of the variance in patient satisfaction is explained by the WAI-SR, *R-square* = .398; *F*_(1, 50)_ = 33.060, *p* < 0.001. WAI-SR partly mediates at least the relationship between group condition and patient satisfaction
19	Gómez Penedo et al. (36)	WAI-I: Working Alliance Inventory for Guided Internet Interventions, C	Post treatment	Task & goal subscale: 3.17 (0.91) Bond subscale: 3.56 (1.15)	Significant effect of the tasks and goals subscale on the estimated PHQ-9 value at the end of follow-up (*B* = −1.74, *SE* = 0.40, 95% CI [−2.52, −0.96], *t*(206) = −4.37, *p* < 0.001)
20a	Hadjistavropoulos et al. (37)	Therapeutic Alliance Questionnaire (TAQ), C	After module 6 and prior to completing module 12	Mid-treatment: 83.47 (13.89) Post-treatment: 83.20 (15.35)	Mid-treatment TAQ scores were not significantly correlated with PHQ-9 post-treatment scores (controlling for pre-treatment PHQ-9 scores)
20b	Hadjistavropoulos et al. (37)	Therapeutic Alliance Questionnaire (TAQ), C	After module 6 and prior to completing module 12	Mid-treatment: 85.82 (10.85) Post-treatment: 86.93 (12.42)	Mid-treatment TAQ scores were not significantly correlated with GAD-7 post-treatment scores (controlling for pre-treatment GAD-7 scores)
21	Klein et al. (39)	Therapeutic alliance questionnaire (TAQ), C	n.a.	89.2 (15.1)	n.a.
22	Duffy et al. (35)	Scale to assess the therapeutic relationship (STAR), C & T	At treatment exit	**STAR-P** (Client's perspective), treatment completers: 37.410 (1.543) **STAR-C** (therapist's perspective), treatment completers: 30.543 (1.500)	STAR-P, client's perspective: Treatment completers showed a significant increase in STAR-P scores of on average 3.9 points from baseline to average end of treatment (day 46) (95% CI [−5.36, 1.26], *t* (82)= −3.195, *p* = 0.002). STAR-C, therapist's perspective: For dropout clients, the STAR-C scores declined significantly by on average 5.4 points from baseline to end of treatment (day 46) (95% CI [2.10, −8.73), *t*(308) = 3.236, *p* = 0.001)
23	Reynolds et al. (46)	Agnew Relationship Measure (ARM), short form, C & T	Weekly	**Client ratings** Bond and partnership: 5.97 (1.26); Confidence: 6.19 (1.24); Openness: 5.27 (1.42) **Therapist ratings** Bond and partnership: 5.72 (.94); Confidence: 5.76 (.99); Openness: 4.73 (1.55)	n.a.

*If a study did not report certain information, this is indicated with n.a. (not available)*.

*The Working Alliance Inventory is from Horvath and Greenberg (24); the Working Alliance Inventory—Short Form is from Busseri and Tyler (50); the Working Alliance Inventory—Short Form Revised is from Hatcher and Gillaspy (51); the Working Alliance Inventory for Guided Internet Interventions is from Penedo et al. (36); the Therapeutic Alliance Questionnaire is from Bickman et al. (52), the Scale to Assess the Therapeutic Relationship is from McGuire-Snieckus et al. (53), the Agnew Relationship Measure is from Agnew-Davies et al. (54)*.

The reviewed studies varied in their chosen points in time for measurements. Six of the studies reviewed here measured the working alliance early on in treatment, specifically in the second or third week of treatment or after the third session. Other studies took measurements after a set number of modules (*n* =1), after the completion of certain modules (*n* =3), after the first half of treatment and post-treatment (*n* =1), and solely after treatment (*n* = 1). If multiple measurements were available, we chose to report early measurements in Table 3, since it is recommended to report early measurements of the therapeutic alliance (55).

#### Therapeutic Relationship Measure and Quality of Therapeutic Relationship

##### The Working Alliance Inventory

Most reviewed studies used a version of the Working Alliance Inventory (WAI) (24) for measurements of the therapeutic alliance (*n* = 19). The WAI has a client and therapist version, of which both scores can be combined to form a composite score. However, frequently only the client version of the WAI is used, as was also the case in the majority of studies included here (*n* = 17). The original WAI has 36 items rated on a 7-point Likert scale, and was used by only one study. Fourteen studies used the 12-item short form of the WAI, the WAI-S. A few studies used a modified version of the WAI-S, adapted for guided internet interventions (*n* = 2) or for online help for women (*n* = 1). In addition, four studies used the revised short form of the WAI (WAI-SR), which also has 12 items but uses a 5-point Likert scale (51). Finally, one study used the WAI-I, which is a new scale developed specifically for guided internet interventions (36), and was derived from the WAI-SR. The WAI-I also has 12 items and uses a 5-point Likert scale.

The WAI and WAI-S items are typically averaged into a total score between 1 and 7 (50). Measurements of the average working alliance with the WAI or the WAI-S ranged from 4.30 (SD: 1.27) to 6.3 (SD: 0.54) across 12 studies that examined 15 interventions. The weighted average score of all WAI and WAI-S measurements was 5.66 with a weighted average standard deviation of 0.84, which indicates a positive working alliance. Only three of the four studies using the revised short form (with a maximum score of 5) of the WAI reported total WAI-SR-scores (see Table 3), with a weighted average of 3.23 (weighted SD: 0.8). Of those, one reported a low mean score of 2.34 (SD: 0.98) (38).

##### Other Measures

Additionally, a few studies used different scales to assess the therapeutic alliance. Two studies that examined three interventions used the Therapeutic Alliance Questionnaire [TAQ (52)], which only assesses the client's perspective. The maximum score for the TAQ is 102, and a score above 80 is considered to reflect a high working alliance (37). The two studies included here showed a weighted average TAQ-score of 85,26 with a weighted average standard deviation of 12.44 (based on the mid-treatment scores when these were included), indicating a good working alliance.

One study used the Scale to Assess the Therapeutic Relationship [STAR (53)], which consists of a separate patient (STAR-P) and clinician (STAR-C) scale, both with 12 items and scores that can range from 0–48, with higher scores indicating a better therapeutic relationship. The study included here reported an average STAR-P score of 37.41 (standard deviation: 1.54) and an average STAR-C score of 30.54 (standard deviation: 1.5).

Finally, one study used a short form of the Agnew Relationship Measure [ARM (54)]. The ARM also has parallel versions for clients and therapists, both with 12 items and a 7-point Likert scale. The study included here only reported the scores on subscales for clients and therapist, and not a composite score. These sub scores ranged from 5.27 to 6.19 (out of 7) for clients, and from 4.73 to 5.76 (out of 7) for therapists, which seems to indicate a positive therapeutic relationship.

#### Relationship Between Therapeutic Relationship and Outcome

Reported findings on the statistical relationships between measurements of the therapeutic alliance and outcome measures of treatment are shown in Table 3. Analysis techniques used included bivariate and partial Pearson correlations, hierarchical multiple regression analysis and multi-level hierarchical linear modeling.

The 23 reviewed articles included 28 interventions. For 22 of these interventions, studies reported statistical relationships between measurements of the therapeutic alliance and outcome measures. Firstly, for 8 out of 13 interventions (8 out of 10 studies), significant correlations were reported between the therapeutic alliance measures and change-scores on one or more primary outcome measures, ranging from small (*n* = 1) to moderate (*n* = 7) and strong (*n* = 1) in magnitude. Further, for 6 out of 7 interventions (5 out of 6 studies) that were examined on the predictive value of the alliance on subsequent treatment outcomes, higher degrees of the therapeutic alliance significantly predicted better treatment outcomes.

Three out of three studies found that the therapeutic alliance significantly predicted the secondary outcome measures of, respectively, compliance (*n* =1) and client satisfaction (*n* = 2). In the case of the influence of the alliance on client satisfaction, the effects found in both studies were strong. Finally, three out of three studies found that subscales of the WAI, such as agreement on tasks, significantly predicted (n = 1), or were significantly and moderately correlated with (*n* = 2) outcome measures of the assessed treatment.

## Discussion

This scoping review aims to summarize the available research on the therapeutic alliance in text-based digital psychotherapy within the last 15 years, in order to enhance its responsible use. A total of 23 articles that examined the therapeutic alliance in 28 text-based interventions were reviewed. These articles were explored on several participant, study and intervention characteristics, as well as the type of measurements of the therapeutic alliance and its relationship to treatment outcome.

In general, our findings show that research on this topic has been conducted with a variety of client groups and treatment modalities. However, most studies focused on clients diagnosed with anxiety and/or depression symptoms and used a form of internet-based cognitive behavioral therapy (ICBT) as modality for treatment delivery. Further, most text-based digital treatments solely used asynchronous communication methods, such as emails, delayed chats, and integrated text-based communication features within websites and platforms.

An essential precondition of the responsible use of digital, text-based psychotherapy is knowing whether a therapeutic alliance can be established in this therapy format, and whether the strength and type of a therapeutic alliance that is established through text is comparable to the one found in face-to-face treatment. The therapeutic alliance scores in the reviewed articles on digital text-based psychotherapy were mostly high, thus suggesting that positive alliances can indeed be established in digital interventions even if only text-based communication modalities are used between clients and therapist. The majority of the reported statistical relationships of the therapeutic alliance showed either significant correlations between the alliance and treatment outcome, or found that the alliance significantly predicted primary and secondary outcome measures.

### Scope of the Reviewed Research

In previous reviews on the therapeutic alliance in digital therapy, a very limited number of studies was found investigating the therapeutic alliance in text-based digital therapy (6, 10). The current review therefore provides an updated overview specifically on the therapeutic alliance in this therapy format. With a number of 23 included studies, of which 7 studies were published from 2017 and onwards [when Berger's narrative review was conducted (10)], the body of evidence on the working alliance in text-based digital treatment seems to be growing. Furthermore, of the included studies in the current review, 14 studies investigated the therapeutic alliance as a primary objective, compared to 6 studies in a review from 2012 (6). With a larger evidence base, the current review confirms indications from earlier reviews of a similar therapeutic alliance in digital therapy and face-to-face therapy and mixed to positive relationships of the alliance with treatment outcomes (6, 10).

Although a range of treatments for various client groups was found in the reviewed studies, most of them concerned (a combination of) anxiety and depression symptoms. This is not surprising, given that anxiety and mood disorders are the most prevalent mental disorders (56). Furthermore, with the great majority of studies reviewed here using a cognitive behavioral therapy approach or framework for internet-based psychotherapy it becomes apparent that this approach also dominates the treatment options online, in line with earlier findings on internet psychotherapy (6, 10). This might be explained by the fact that the short-term interventions and techniques that are commonly used in CBT fit well in an online format (57), and may also be more easily integrated into internet-based psychotherapy given the length of the treatments reviewed here (5–16 weeks). Other psychological approaches such as psychodynamic approaches or third-wave CBT were underrepresented in the current review. While internet-based treatments based on psychodynamic theory and third-wave approaches are slowly starting to appear, the working alliance or similar constructs relating to the therapeutic relationship have yet to be researched (58, 59).

Finally, the reviewed studies examined various digital treatment options, but it is worth mentioning that the authors of the studies often originate from the same research groups from Germany and Sweden. It seems that mainly these groups investigate the therapeutic alliance online, which suggests a lack of variety in researchers studying the subject, and shows that research interest in this topic is not yet widespread. Furthermore, since the country in which studies on (digital) psychotherapy are conducted likely influence the generalizability of the results, caution is necessary when interpreting these findings. The current conclusions may be limited to the North-European context and culture and may not be representative for other countries and cultures.

### The Therapeutic Alliance and Responsible Digital Treatment

Our findings generally show high levels of therapeutic alliance in text-based digital psychotherapy, comparable to those reported for face-to-face treatment (50). Therefore, the establishment of a good therapeutic relationships seems to be possible independent of the medium (digital text-based or face-to-face). Additionally, the majority of the included studies show significant and positive relations between the therapeutic alliance and primary or secondary outcome measurements, such as a strong relationship with client satisfaction. The formation of a good therapeutic alliance, especially when related to better treatment outcomes, supports the notion that text-based digital treatments can be a responsible addition or alternative to face-to-face treatment or (long) waiting lists.

#### Quality of the Therapeutic Alliance and Alliance-Outcome Relationship

To measure the therapeutic alliance, most of the reviewed studies (*n* = 10) used either the Working Alliance Inventory [WAI (24)] or its short form [WAI-S (50)]. The scores on the WAI and WAI-S were generally high with a combined average score of 5.66 (out of 7). This indicates a good working alliance that is comparable to ratings in face-to-face treatment, such as the mean of 5.87 found in a study on the Working Alliance Inventory in face-to-face treatment (50). In addition, four studies used other measures of the working alliance. All of the reported scores for these measures also indicated similarly high scores of the working alliance, (far) above the midpoints of the different scales.

Only one of the reviewed studies (38) reported a low mean score of 2.34 for the revised short form of the WAI (the WAI-SR, with a maximum score of 5). Nonetheless, additionally reported scores by the researchers did indicate an increase in working alliance ratings by week 9. The study was unique in their targeted population of clients diagnosed with chronic tinnitus and their sample of mainly male clients. Given the lack of research on online treatment with this client group it is unclear whether the relatively low score and late increase in alliance quality stems from client characteristics, the specific treatment or other factors.

With respect to the statistical relationship between measurements of the working alliance and outcome measures of treatment the results were somewhat varied. Many studies indicated significant moderate [e.g., (31)] correlations of the total working alliance scores or subscales scores with outcome measures. One study found small correlations (8) and another found strong correlations (47), while a few studies did not find significant correlations with primary outcome measures [e.g., (28)]. Moreover, several studies indicated that early measurements of the working alliance significantly predicted better treatment outcomes or secondary outcomes such as compliance to treatment or client satisfaction.

The positive relationship between the therapeutic alliance and treatment outcomes indicates that the type of alliance that emerges in text-based digital psychotherapy is indeed beneficial to the therapeutic work. The finding that many studies found a significant and moderate relationship between the alliance and treatment outcomes is also in accordance with a previous meta-analysis on the alliance-outcome association in digital psychotherapy that found a significant overall correlation of *r* = 0.28 (15), as well as a more recent meta-analysis on the same topic that found a significant overall correlation of *r* = 0.20 (19). Since the research on this topic was until recently very limited (10), our review provides the necessary update on the working alliance in this evolving and growing field. Hopefully, our findings can serve to give therapists more confidence in their abilities to develop a functional working alliance in internet-based psychotherapy, since earlier studies showed that this confidence is often lacking in therapists (7, 17).

#### What Knowledge Is Missing?

Especially for clients diagnosed with anxiety, depression, or PTSD symptoms and digital text-based treatments based on CBT, our review shows evidence that good therapeutic alliances can be established in text-based psychotherapies. Here, the alliance does not seem to be compromised due to the communication being text-based instead of face-to-face. With regard to other client groups and treatment approaches, the research on the therapeutic alliance in digital text-based psychotherapy is still in its infancy. This means that we should be careful with generalizing our findings from the current review to other client groups and treatment approaches. However, since we did not find any obvious differences for client groups or different treatment forms, we expect that the working alliance in text-based digital treatment works similarly across the board, in line with findings on internet-based treatment in general (15).

Additionally, to judge whether digital text-based treatments are a responsible option, measuring the quality of the therapeutic alliance solely quantitatively with the WAI could reflect a rather narrow view of the alliance. The therapeutic alliance is a dynamic construct, that fluctuates over time and that a therapist should always closely guard during the therapy process (20), whereas in most of the included studies the working alliance was only measured at one moment in time. A different research approach, such as experience sampling (60), could therefore be a suitable addition to research on the therapeutic alliance. Experience sampling could also give more insight into the naturally occurring ruptures and the corresponding repair work in the therapeutic relationship. These are not taken into account in a scale such as the WAI, even though ruptures seem likely to occur in relationships that are formed on the basis of reduced communication cues and responsiveness (10). It is possible that other important, more complex elements in the relationship are currently missed as well, such as self-disclosure or empathy (61).

An essential value in mental health care, for example, is the compassion between the therapist and the client (62, 63). Compassion consists of different elements: recognizing suffering, understanding its universality, emotional resonance, tolerating uncomfortable feelings, and the motivation to act to alleviate suffering (64). The presence of compassion in treatment has many benefits (65), and it can strengthen the therapeutic alliance (66). However, the ability to express and transfer compassion in digital treatments and therapeutic (text-based) relationships has not been examined yet, and no scale exists to measure compassion in digital treatment forms.

The WAI, for instance, consists of three subscales, measuring the agreement between client and therapist on goals and tasks, as well as the bond (16, 24). This final subscale, the bond, comes closest to measuring elements of compassion in treatment, but its items are limited to the mutual liking, respect and appreciation in the relationship. These items do not do the comprehensive concept of compassion justice. Moreover, the WAI stems from 1989 (24), and was not developed with digital treatment options in mind. Therefore, to examine if a fundamental value such as compassion does not get lost in digital, text-based treatment as compared to face-to-face treatment, a new scale to measure compassion in these treatment forms is needed.

### Study Limitations

There are a few potential limitations concerning the results of this review. Firstly, the choice for a scoping review was made with the aim for a broad coverage of the topic. As is characteristic of scoping reviews, the current scoping review did not appraise the methodological quality of the reviewed articles. Secondly, regarding the used search strategy, search terms such as “online psychotherapy” and “online mental health” were chosen in an attempt to achieve a broad coverage and to not steer in the direction of certain mediums. Since the included interventions are mostly web-based, it is possible that we missed some studies that included other forms of digital text-based psychotherapy, such as via text-messaging or via apps. However, a search including additional search terms such as “SMS,” “text-messaging” and “apps” shows that many interventions using these mediums pertain to unguided, self-help interventions and not guided psychotherapy.

Finally, abstracts included in this review were required to report the assessment of the working alliance (or therapeutic alliance or therapeutic relationship). It is possible that studies did not report their measurements of the working alliance in the abstract, and were therefore not included in this review. These limitations could be addressed in future reviews to provide an even more extensive synthesis of findings on the working alliance online.

### Directions for Future Research

This review can be seen as a step toward a more positive perspective on relationships between clients and therapists in digital treatment and highlights the fact that high quality working alliances can be established digitally, and through text. Of course, this also raises new questions, such as to what extent this good digital therapeutic relationship is related to the therapist, and how much of it is mediated by the digital interaction. Moreover, future research could assess qualitatively how client and therapist interact online to establish this valuable relationship, since it remains unclear what the content is of their communication that has led to a good therapeutic alliance. This knowledge is critical to understand what helps build the therapeutic alliance, and could have practical implications for clients and therapists considering the use of text-based digital interventions. A more in-depth insight into the therapeutic relationship could also give more clarity on any elements that current measures might miss, such as the presence of compassion. This might give us some understanding of what type of behavior can enable a positive working alliance and, possibly, how digital treatment options and communication modalities can facilitate these behaviors.

Furthermore, the number of studies and interventions included in the current systematic scoping review shows that it is possible to conduct a meta-analysis on this topic. Such an analysis could provide a richer picture of the role of the therapeutic alliance in text-based digital psychotherapy, for example by evaluating which types of platform and forms of communication enable better therapeutic alliances and to what extent the alliance relates to the treatment outcome based on effect sizes.

Although the generalizability of the present results must be established in future research, and more comprehensive measurements of the therapeutic alliance are needed, this scoping review provides support for the possibility of a good working alliance in various text-based digital psychotherapeutic treatments and with various groups of people *diagnosed* with different mental disorders. It might inspire clinical psychologists, psychiatrists and mental health care workers to consider accessible internet- based options with a low-threshold as an addition or alternative to face-to-face treatment.

## Author Contributions

LJ and MLN conceptualized the study. CML and LJ reviewed the literature and led the paper drafting. All authors edited multiple drafts and reviewed the final paper.

## Conflict of Interest

The authors declare that the research was conducted in the absence of any commercial or financial relationships that could be construed as a potential conflict of interest.
